# Obesity Downregulates MicroRNA-126 Inducing Capillary Rarefaction in Skeletal Muscle: Effects of Aerobic Exercise Training

**DOI:** 10.1155/2017/2415246

**Published:** 2017-03-06

**Authors:** João Lucas Penteado Gomes, Tiago Fernandes, Ursula Paula Reno Soci, André Casanova Silveira, Diego Lopes Mendes Barretti, Carlos Eduardo Negrão, Edilamar Menezes Oliveira

**Affiliations:** School of Physical Education and Sport, University of Sao Paulo, Sao Paulo, SP, Brazil

## Abstract

*Background.* We investigated the effects of exercise training (ET) on miR-126 levels and skeletal muscle angiogenesis in obese Zucker rats.* Results.* Zucker rats were randomly assigned to sedentary and swimming-trained groups: lean sedentary (LS) and trained (LTR); obese sedentary (OB) and trained (OBTR). The OB group displayed capillary rarefaction compared with the LS group. In contrast, ET increased the capillary/fiber ratio by 38% in the LTR group and normalized capillary rarefaction in the OBTR group. VEGF, PI3K, and eNOS levels were reduced in the skeletal muscle of the OB group. ET normalized VEGF, PI3K, and eNOS levels in OBTR, contributing to vascular network homeostasis. PI3KR2 inhibits PI3K, a key mediator of the VEGF signaling pathway. Obesity decreased miR-126 and increased PI3KR2 levels compared with the LS group. However, ET normalized miR-126 levels in the OBTR group versus the LS group and decreased expression of PI3KR2.* Conclusion.* Our findings show that obesity leads to skeletal muscle capillary rarefaction, which is regulated by decreased miR-126 levels and increased PI3KR2. Inversely, ET normalizes miR-126 levels and VEGF signaling and should be considered an important therapeutic strategy for vascular disorders.

## 1. Introduction

Obesity is a chronic disease caused by an excess of body fat; this leads to a number of systemic body changes that predispose to the development of other diseases such as diabetes, hypertension, dyslipidemia, and cancers. The obesity epidemic has continues to increase and now affect about 13% of the global population and is a major global public health problem [1–3].

Obesity causes deleterious morphological changes in skeletal muscle, for example, capillary rarefaction, which often occurs in vascular diseases caused by inflammation and an abnormal lipid profile [4–6]. In addition, obesity alters the content of intramuscular fat and deregulates the expression of several proteins related to angiogenesis and trophism [7–9]. In contrast, exercise training (ET) is an important nonpharmacological therapeutic strategy for the treatment of chronic diseases. ET promotes beneficial morphological changes in many tissues and body systems, such as an increase in capillary density, increased diameter of muscle fibers, and a reduction in intramuscular fat content [9–12]. However, the effects of ET on the molecular mechanisms of obese skeletal muscle capillary rarefaction are poorly understood.

The microRNAs (miRs) have been widely studied as regulators of gene translation and thus protein content. MiRs are small noncoding RNA molecules (containing about 17~22 nucleotides) that exert functions such as RNA silencing and posttranscriptional regulation of gene expression. These effects occur by miR coupling to mRNA in the 3′ untranslated seed region, thereby preventing its translation [13–15].

MiR-126 controls angiogenesis and regulates the formation, survival, and maintenance of new vessels [16–18]. Phosphatidylinositol 3-kinase regulatory subunit beta (PI3KR2) is one of the main targets of miR-126 and controls the vascular endothelial growth factor (VEGF) signaling pathway via direct inhibition of phosphatidylinositol 3-kinase (PI3K *α*-110). Thus, miR-126 inhibits PI3KR2 and promotes angiogenesis through an indirect increase in PI3K and VEGF. Through this process, the expression of proteins in the nitric oxide synthase (eNOS) pathway is activated, which also enhances angiogenesis. Knockdown of miR-126 in animals leads to impaired vascular formation due to a reduction in the migration of endothelial cells during the genesis of vessels; this compromises blood vessel integrity and leads to hemorrhage in the embryonic period [18].

ET regulates the expression of various miRs that regulate biological processes related to adaptation [17, 19]. The expression of miR-126 increases in healthy trained rats [19] and is severely decreased in rats with chronic diseases such as hypertension [17]. However, ET has been shown to restore miR-126 levels in the skeletal muscle of hypertensive rats and induces angiogenesis [17]. However, the effects of ET in terms of controlling the expression of miRs in skeletal muscle in the context of obesity need further investigation. In the present study, we found that reduced miR-126 levels induced capillary rarefaction in skeletal muscle by targeting PI3KR2 in obese Zucker rats. ET counteracted this effect by restoring the expression of miR-126 and skeletal muscle capillarity in obese Zucker rats.

## 2. Methods

### 2.1. Experimental Group

Twenty-one Zucker rats were randomly divided into four groups: sedentary lean (LS, *n* = 5), trained lean (LTR, *n* = 5), obese sedentary (OB, *n* = 5), and obese trained (OBTR, *n* = 6). The animals were housed 3 to 5 per cage in a room heated between 22°C and 24°C and were on an inverted light-dark cycle; water and food were given ad libitum. After the training protocol, animals were euthanized and the relevant tissues were dissected and weighed. All procedures were performed according to the Ethical Principles of Animal Experimentation and the approved by the Ethics Committee on Animal Use at the University of Sao Paulo.

### 2.2. Swimming Training Protocol

Swimming training was performed according to the protocol developed by Medeiros and colleagues [20] in a swimming system with water at 30–32°C. The training duration was 10 weeks with 5 weekly sessions with a progressive increase in time, reaching 60 minutes, and with a progressive increase in workload reaching 4% of the body weight of the animal.

### 2.3. Quantification of Intramuscular Lipids by Oil Red O

Serial sections of skeletal muscle (soleus) were prepared at a thickness of 10 *µ*m. Muscle sections were fixed in 3.7% formaldehyde and stained with Oil Red O working solution (500 mg of Oil Red O added to 100 ml of aqueous solution with 60% triethyl phosphate (Fluka) and 8 ml of deionized water). The final product of this procedure is red-stained lipids. The intramuscular fat content was analyzed in the soleus muscle in intact cellular membranes with a computer-assisted morphometric system (Leica Quantimet 500, Cambridge, UK) able to measure the red wavelength. For each animal, approximately 10 visual fields were analyzed.

### 2.4. Oxygen Uptake Measurements

Oxygen uptake (O_2_) was measured by expired gas analysis during graded treadmill exercise training. Gas analysis was performed using an oxygen and carbon oxide analyzer (Sable Systems SS3, FC-10a O_2_/CO_2_ analyzer, Las Vegas, NV). O_2_ was calculated using the measured flow through the metabolic chamber, the expired fraction of effluent oxygen, and the fraction of oxygen in room air.

### 2.5. Skeletal Muscle Oxidative Enzyme Activity

Citrate synthase activity, used as an index of aerobic ET, was determined spectrophotometrically in mixed soleus muscle [21]. The enzyme activity was measured in soleus muscle homogenates, and the amount of the complex resulting from acetyl-CoA and oxaloacetate was determined at 412 nm and 25°C at an interval of 10 min. The solubilized protein extracts from the homogenates were quantified in duplicate using bovine albumin as the standard. Citrate synthase activity was then normalized to the total protein content and reported in nanomoles per milligram of protein per minute.

### 2.6. Capillary-to-Fiber Ratio

Twenty-four hours after the last exercise training session, sedentary and trained rats were killed and the soleus muscle was immediately frozen in melted isopentane and then stored in liquid nitrogen. Frozen muscle was cut into 10 *μ*m cross-sections from the proximal to the distal region using a cryostat (Micron HM505E; Zeiss, Walldorf, Germany). Muscle sections were then probed for myofibrillar ATPase activity with alkaline medium (myosin ATPase, pH 10.3) or acid medium preincubation (myosin ATPase, pH 4.6). The myosin ATPase reaction was used to identify muscle fibers and capillaries. The capillary-to-fiber ratio was quantified using a 10 × 10 grid optically superimposed on each of 10 nonoverlapping fields at ×200 magnification, distributed in a random manner using a computer-assisted morphometric system (Quantimet 500; Leica, Cambridge, UK). To calculate the capillary-to-fiber ratio, the total number of capillaries was divided by the total number of fibers counted in the same field. Only vessels with a diameter < 10 *μ*m were counted. All analyses were conducted by a single observer (J. Gomes) blinded to rat identity.

### 2.7. Analysis of miR-126

miR-126 expression was measured using real-time PCR and the TaqMan MicroRNA Assay (Applied Biosystems, CA, USA). The 20 *μ*l PCR reaction included 1.33 *μ*l RT product, 10 *μ*l TaqMan Universal PCR master mix II (2x), 7.67 *μ*l nuclease-free water, and 1 *μ*l of primers and probe mix from the TaqMan MicroRNA Assay for miR-126 (INV 2228). The reactions were incubated in a 96-well optical plate at 95°C for 10 min, followed by 40 cycles of 95°C for 15 s and 60°C for 1 min. Samples were normalized by evaluating U6 expression. Each sample was analyzed in triplicate. Relative quantities of target gene expression in sedentary rats versus trained rats were compared after normalization to the values of the reference gene (ΔCT). Fold changes in miRNA expression were calculated using the differences in ΔCT values between the two samples (ΔΔCT) and the equation 2^−ΔΔCT^. Results were expressed as % of control.

### 2.8. Immunoblotting

The protein levels PI3KR2, PI3K, VEGF, and eNOS in the soleus muscle were analyzed by Western blotting. The frozen soleus muscle (100 mg) was homogenized in cell lysis buffer containing 100 mM Tris-HCl, 50 mM NaCl, 1% Triton X-100, and a protease and phosphatase inhibitor cocktail (1 : 100; Sigma-Aldrich, MO, USA). Soleus tissue debris was removed by centrifugation at 3,000 ×g, 4°C, 10 min. Samples were loaded and subjected to SDS-PAGE on polyacrylamide gels (6–15%) depending on the protein molecular weight. After electrophoresis, proteins were electrotransferred to a nitrocellulose membrane (BioRad Biosciences, NJ, USA). Equal loading of samples (30 *μ*g) and even transfer efficiency were monitored with the use of 0.5% Ponceau S staining of the blot membrane. The blot membrane was then incubated in a blocking buffer (5% nonfat dry milk, 10 mM Tris-HCl (pH 7.6), 150 mM NaCl, and 0.1% Tween 20) for 2 h at room temperature and then incubated overnight at 4°C with rabbit anti-VEGF (Abcam, Cambridge, UK ab46154), anti-PI3K (Abcam, Cambridge, UK ab151549), anti-PI3KR2 (Abcam, Cambridge, UK ab62901), an anti-eNOS (Cell Signaling Tech, MA, USA #9572) polyclonal antibodies, and a mouse anti-GAPDH monoclonal antibody (Abcam, Cambridge, UK ab9484). Binding of the primary antibody was detected by peroxidase-conjugated secondary antibodies and enhanced chemiluminescence reagent (Amersham Biosciences, NJ, USA) and detection was performed in a digitalizing unit (ChemiDoc; BioRad, CA, USA). The bands were analyzed using Image J software (ImageJ Corporation based on NIH image). Skeletal muscle GAPDH expression levels were used to normalize the results, which are expressed as a percentage of control expression.

### 2.9. Statistical Analysis

The results are represented as mean ± SEM. Statistical analysis was performed using two-way ANOVA. *p* values < 0.05 were accepted as statistically significant. The Duncan post hoc test (STATISTICA software; StatSoft, Tulsa, OK) was used for individual comparisons between means when a significant change was observed with ANOVA. The Pearson coefficient of correlation was used to analyze the correlations between parametric data.

## 3. Results

### 3.1. Experimental Design

**Figure 1:**
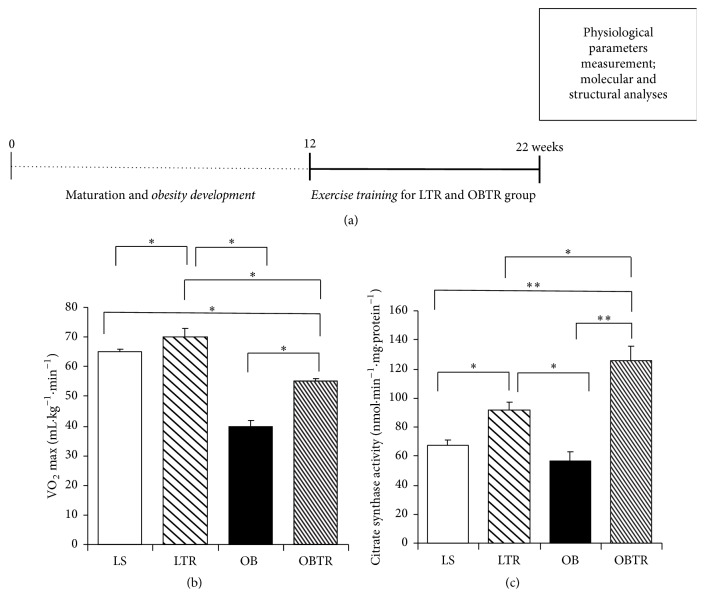
(a) Presentation of experimental design, (b) VO_2_ max (ml·kg^−1^·min^−1^), and (c) citrate synthase activity (nmol·min^−1^·mg·protein^−1^). Groups: LS: lean sedentary; LTR: lean trained; OB: obese sedentary; OBTR: obese trained. Data are reported as means ± SEM. ^*∗*^*p* < 0.05; ^*∗∗*^*p* < 0.01.

All animals remained sedentary from 0 to 12 weeks to allow obesity development and maturation. After 12 weeks, animals (LTR and OBTR groups) were submitted to the ET protocol for 10 weeks, while the other groups remained sedentary. At 22 weeks, all the physiological parameters (cardiac frequency, blood pressure, and VO_2_) were measured; there were no differences in these parameters between groups. All animals were killed after the last ET session at 22 weeks. All tissues were adequately collected and the molecular and structural analyses were subsequently performed (Figure 1(a)).

### 3.2. Effectiveness of Aerobic Exercise Training

To confirm the effectiveness of ET, we evaluated the oxygen consumption peak (VO_2_ max). At the end of the protocol, the OB and OBTR groups had lower oxygen uptake compared with the LS group. However, the OBTR group showed a higher VO_2_ max compared with the OB group. VO_2_ max was significantly higher in the LTR group compared with all other groups (LS: 65.5 ± 1.2 LTR 70.1 ± 3.8; OB: 40.5 ± 2.15; and OBTR: 55.3 ± 1.3 mL/kg/min) (Figure 1(b)). We also evaluated oxidative citrate synthase enzyme activity in the soleus muscle as an aerobic ET marker. The results show that the OB group was not different from the LS group; however, the OBTR and LTR groups showed a significant increase in enzyme activity compared with the LS and OB groups, demonstrating the effectiveness of the ET protocol (LS: 67 ± 4.35; LTR: 92 ± 4.65; OB: 57 ± 5.7 and OBTR: 126 ± 9.48 nmol·min^−1^·mg^−1^) (Figure 1(c)).

### 3.3. Effect of Exercise Training on Body Composition

**Figure 2:**
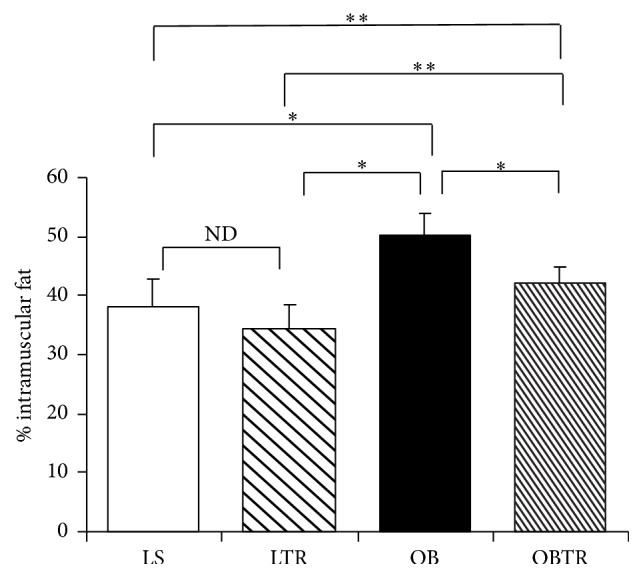
Percentage of intramuscular fat. Groups: LS: lean sedentary; LTR: lean trained; OB: obese sedentary; OBTR: obese trained. Data are reported as means ± SEM. ^*∗*^*p* < 0.05; ^*∗∗*^*p* < 0.01; ^ND^*p* > 0.05.

The results show that the OB group had a higher content of intramuscular fat compared with all other groups. In contrast, ET normalized the intramuscular fat content in the OBTR compared to the LS group. The LTR group was not different from the LS group. Thus, the data indicate that ET prevented the accumulation of intramuscular fat only in the OBTR group (LS: 37.93 ± 3.73; LTR: 34.55 ± 4.97; OB: 50.08 ± 2.89; and OBTR: 42.14 ± 3.93) (Figure 2). In addition, we have previously shown that body weight and visceral fat is greatly increased in OB group, but ET is able to attenuate the increase in OBTR body weight and visceral fat [10].

### 3.4. Angiogenesis and VEGF Protein Expression

**Figure 3:**
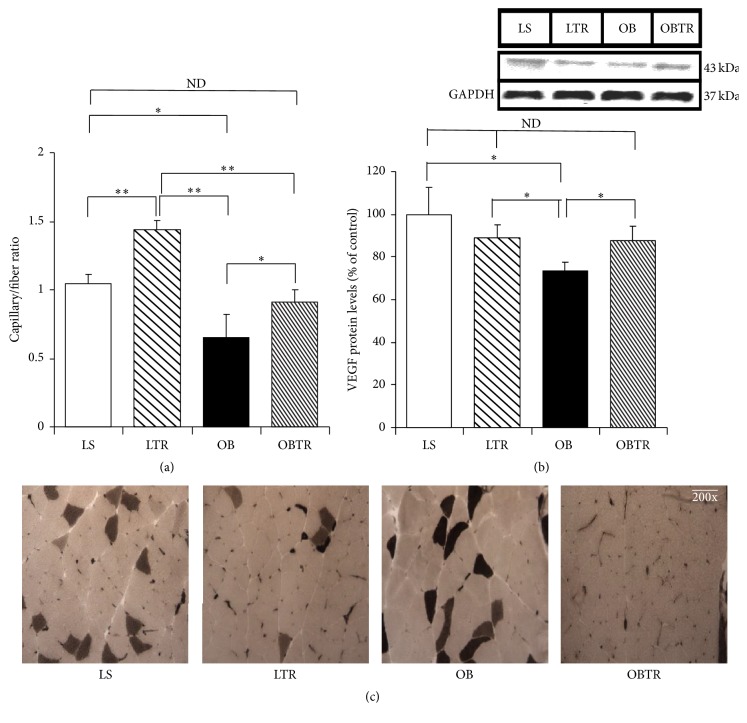
(a) Percentage of capillary/fiber ratio; (b) VEGF protein levels; (c) representative images of capillary density. Groups: LS: lean sedentary; LTR: lean trained; OB: obese sedentary; OBTR: obese trained. Data are reported as means ± SEM. ^*∗*^*p* < 0.05; ^*∗∗*^*p* < 0.01; ^ND^*p* > 0.05.

Our data show that the OB group presented severe capillary rarefaction, showing 37% less capillarity compared to control. In contrast, ET normalized capillary rarefaction in the OBTR group, suggesting that increased muscle contractions may increase angiogenesis. In addition, the LTR group presented a 38% increase in capillarity compared to LS (Figure 3(a)) (LS: 1.05 ± 0.08; LTR: 1.44 ± 0.07; OB: 0.65 ± 0.17; and OBTR: 0.91 ± 0.09).

VEGF is a potent angiogenic factor. VEGF protein expression was decreased in the OB group compared to the LS group. However, ET normalized VEGF expression in the OBTR group toward control levels, indicating the ET is a regulator of angiogenic factors in obesity. Interestingly, there was no change in the expression of VEGF protein in the LTR group compared to control (Figure 3(b)) (LS: 100 ± 12.5; LTR: 89.1 ± 6.37; OB: 73.2 ± 4.66; and OBTR: 87.5 ± 6.81).

### 3.5. Effect of Exercise Training and Obesity on miRNA-126 Expression and Relation with Angiogenesis

**Figure 4:**
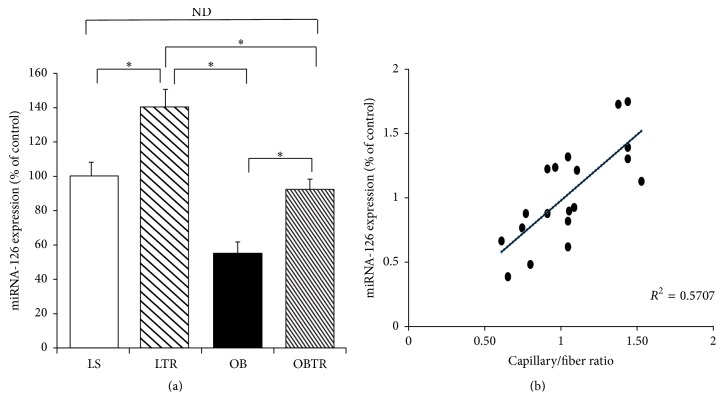
(a) Expression of miRNA-126 by real-time PCR and (b) positive correlation between miRNA-126 expression and capillary–fiber ratio. Groups: LS: lean sedentary; LTR: lean trained; OB: obese sedentary; OBTR: obese trained. Data are reported as means ± SEM. ^*∗*^*p* < 0.05; ^ND^*p* > 0.05.

Our data show that miR-126 expression was reduced in the OB group compared with all the other groups. In contrast, ET restored miR-126 expression in the OBTR group toward control levels. Furthermore, ET was also effective in increasing the expression of miR-126 in the LTR group (LS: 100 ± 13.1; LTR: 140 ± 10.7; OB: 55 ± 6.8; and OBTR: 92 ± 6.4). Indeed, our data show that there is a correlation between miR-126 expression and the capillary/fiber ratio. In this study, an increase in the expression of miR-126 was accompanied by increased capillarity (Figure 4(b)).

### 3.6. Effect of Exercise Training and Obesity on PI3KR2 and the PI3K/eNOS Pathway

**Figure 5:**
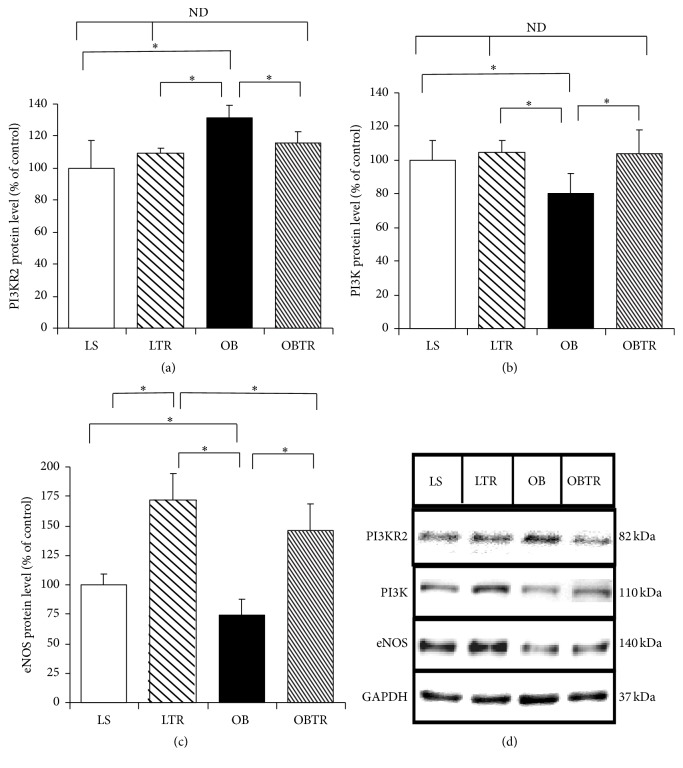
(a) PI3KR2 protein level, (b) PI3K protein expression, (c) eNOS protein expression, and (d) representative blots of PI3KR2, PI3K, and eNOS. Groups: LS: lean sedentary; LTR: lean trained; OB: obese sedentary; OBTR: obese trained. Data are reported as means ± SEM. ^*∗*^*p* < 0.05; ^ND^*p* > 0.05.

PI3KR2 is a target gene of miR-126. PI3KR2 was increased in the OB group compared to the LS group. In contrast, ET restored PI3KR2 in the OBTR group compared to the LS group, demonstrating that ET is able to normalize the expression of this protein. Interestingly, PI3KR2 levels were not different in the LTR group compared to control (Figure 5(a)) (LS: 100 ± 12.5; LTR: 109 ± 3.81; OB: 131.4 ± 7.63; and OBTR: 115.5 ± 7.07).

PI3K protein expression was decreased in the OB group compared to the LS group. In contrast, the OBTR group presented normal levels of this protein, similar to the LS group (LS: 100 ± 11.8; LSTR 105 ± 6.9; OB: 80.2 ± 11.5; and OBTR: 103.7 ± 13.9). Thus, there is a correlation between these two proteins (PI3KR2 and PI3K), where increased expression of PI3KR2 leads to a reduction in the expression of PI3K in obese Zucker rats; ET has the opposite effect (Figure 5(b)).

Our data show that the expression of eNOS protein was reduced in the OB group compared to control, while eNOS expression was normalized in the OBTR group compared to LS group. Moreover, the LTR group had much higher expression of this protein compared to the LS group (LS: 100 ± 9.3; LTR: 172.1 ± 22.1; OB: 74 ± 13.4; and OBTR: 146.6 ± 22.4) (Figure 5(c)).

## 4. Discussion

**Figure 6:**
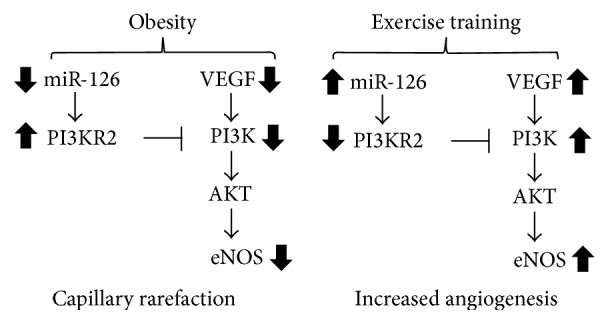
Schematic presentation of swimming training effects on skeletal muscle angiogenesis by miRNA-126. Obesity reduces the miRNA-126 expression leading an increase of PI3KR2, blocking the VEGF pathway, thereby contributing to the phenotype of capillary rarefaction in skeletal muscle. However ET increases miRNA-126 expression improving the VEGF pathway promoting angiogenesis.

Obesity results in many deleterious changes in skeletal muscle, such as capillary rarefaction [9]. Our study suggests that long-term swimming training has a major influence on muscle phenotype in obese Zucker rats. Corroborating with the literature, our study shows that ET is an important nonpharmacological strategy to reduce microvascular rarefaction induced by obesity. The possible molecular factors that precede this impaired phenotype in obesity may include the expression of miR-126 and proteins of the VEGF pathway. The results of this study indicate that miR-126 is downregulated in the skeletal muscle of obese rats and this change was associated with capillary rarefaction. Moreover, we also showed that ET normalizes the expression of miR-126 and reverses the capillary loss caused by obesity (Figure 6).

miR-126 is an endothelial specific miR (angiomiR) and its expression is closely linked to angiogenesis. Expression of miR126 is enriched in endothelial cells and endothelial progenitor cells. This miRNA is involved in the induction of signaling of the embryonic vasculogenesis, being related to a differentiation of embryonic stem cells in endothelial cells and endothelial progenitor cells, promoting their maturation [22]. Knockdown of miR-126 expression in animals results in an impairment in the formation and maintenance of blood vessels and partial embryonic lethality [7].

The miR-126 contributes to vascular homeostasis in mature endothelial cells, associated with maintaining vascular integrity and inhibiting proliferation and motility, in this case actins as an angiogenesis suppressor. However, in cases of vascular lesion, hypoxia or stress on the vessel wall, there is an increase in the expression of miR-126, which activates endothelial cells and endothelial progenitor cells, which contributes to vascular healing and the formation of new vessels [22].

We were the first to show that sedentary obese animals present a decrease in miR-126 expression, which contributes to microvascular rarefaction. However, in other chronic diseases, such as diabetes mellitus II, it has previously been demonstrated that there is also a decrease in the expression of miR-126 in the circulation of rats and humans. Moreover, there is a correlation between the decrease of mir-126 and endothelial apoptosis. It is important to note that a time course study in a type 2 diabetic rat model shows that the gradual decrease of miR-126 and other angiomiRs precedes endothelial apoptosis and decreased vascular integrity, as well as changes in the density of the microvasculature [23].

ET in turn increases blood flow causing a shear stress in the vessel wall; this becomes important because the hemodynamic changes lead to different expressions of microRNAs in the vascular system [24]. The literature demonstrates that only a prolonged ET session in humans leads to increased expression of miR-126 in the circulation [25]. Our previous studies have shown that, in healthy rats, ET increases cardiac miR-126 expression, promoting cardiac angiogenesis [19]. Other studies have shown that ET restores the expression of miR-126 in other diseases [17]. Our evidence suggests that miR-126 also influences control of the microvasculature in the skeletal muscle of obese Zucker rats. Our study shows, for the first time, that ET restores miR-126 expression in the skeletal muscle of obese Zucker rats and is involved in exercise-induced angiogenesis.

PI3KR2 is one of the validated targets of miR-126 and is an important factor in the regulation of angiogenesis. PI3KR2 functions as an indirect negative regulator of VEGF by inhibiting the expression of PI3K. The negative regulation of VEGF by PI3KR2 occurs by the following mechanism: VEGF induces phosphorylation of PI3K; this then generates a phosphorylation cascade that activates AKT, which plays an important role in inducing eNOS signaling, a fundamental process in vessel growth and survival [6, 17]. PI3KR2 inhibits PI3K by inhibiting that phosphorylation cascade; consequently, angiogenesis is inhibited [26, 27].

We show here that obesity decreased miR-126 expression, which increased the protein content of its target genes. Decreased miR-126 led to increases PI3KR2 protein expression in obese rats. However, training restored the expression of miR-126 and consequently normalized PI3KR2 expression in obese rats.

The present study also shows severe capillary rarefaction in sedentary obese animals. Decreased vascularity leads to an inadequate supply of oxygen and nutrients to the tissues and may even cause microvascular necrosis and apoptosis [4, 14, 28]. However, ET has been shown to be a potent therapeutic agent for various vascular diseases. It is known that ET increases microvascular network, caliber, and blood vessels compliance [16, 27, 29].

There are several regulatory proteins involved in angiogenesis; the principal mediators are VEGF and eNOS. Recent findings point to several angiogenic factors that may be upregulated by ET. These factors are influenced by increasing mechanical contraction and shear stress in vessels [17, 19, 27].

We highlight VEGF and eNOS because the expression of these proteins is closely related to ET [17, 19]. They are involved in the proliferation, migration and differentiation of endothelial cells and the formation of new vessels [16, 17, 19, 27]. An increase in eNOS expression leads to an increase in the expression of nitric oxide (NO). These two factors are critical to vasodilation, thereby maintaining the structure, function, and integrity of vessels [19, 27]. VEGF is an important regulator of angiogenesis by promoting the formation of new vessels from other existing vessels; it is also an antiapoptotic factor that prevents capillary loss [17, 27]. There are other proteins that regulate the function of these angiogenic factors, such as PI3KR2, PI3K, and AKT, which are also regulated by aerobic ET [19].

In the present study, we found that obese animals had a deficiency in the expression of the above-mentioned proteins, leading to severe capillary rarefaction in skeletal muscle. ET was able to restore the impaired expression of these proteins and thus partially restored the microvascular network.

In conclusion, miR-126 plays an important role in the control of angiogenic pathways by targeting the expression of PI3KR2. Obesity downregulates the expression of miR-126, which consequently prevents the expression of several angiogenic proteins. However, aerobic exercise training is able to normalize the expression of miR-126 and consequently restore the function of various proteins controlled by it.

## References

[1] Akbartabartoori M., Lean M. E. J., Hankey C. R. (2008). The associations between current recommendation for physical activity and cardiovascular risks associated with obesity. *European Journal of Clinical Nutrition*.

[2] Kelly T., Yang W., Chen C.-S., Reynolds K., He J. (2008). Global burden of obesity in 2005 and projections to 2030. *International Journal of Obesity*.

[3] WHO (2000). *Obesity: Preventing and Managing the Global Epidemic. Report of a WHO Consultation*.

[4] Conway E. M. (2003). Angiogenesis: a link to thrombosis in athero-thrombotic disease. *Pathophysiology of Haemostasis and Thrombosis*.

[5] Frisbee J. C. (2003). Remodeling of the skeletal muscle microcirculation increases resistance to perfusion in obese Zucker rats. *American Journal of Physiology—Heart and Circulatory Physiology*.

[6] Frisbee J. C., Samora J. B., Peterson J., Bryner R. (2006). Exercise training blunts microvascular rarefaction in the metabolic syndrome. *American Journal of Physiology—Heart and Circulatory Physiology*.

[7] Ferraro E., Giammarioli A. M., Chiandotto S., Spoletini I., Rosano G. (2014). Exercise-induced skeletal muscle remodeling and metabolic adaptation: Redox signaling and role of autophagy. *Antioxidants & Redox Signaling*.

[8] Goodpaster B. H., He J., Watkins S., Kelley D. E. (2001). Skeletal muscle lipid content and insulin resistance: evidence for a paradox in endurance-trained athletes. *Journal of Clinical Endocrinology and Metabolism*.

[9] Torgan C. E., Brozinick J. T., Kastello G. M., Ivy J. L. (1989). Muscle morphological and biochemical adaptations to training in obese Zucker rats. *Journal of Applied Physiology*.

[10] Barretti D. L. M., Magalhães F. D. C., Fernandes T. (2012). Effects of aerobic exercise training on cardiac renin-angiotensin system in an obese zucker rat strain. *PLOS ONE*.

[11] Oh K. S., Kim M., Lee J. (2006). Liver PPAR*α* and UCP2 are involved in the regulation of obesity and lipid metabolism by swim training in genetically obese db/db mice. *Biochemical and Biophysical Research Communications*.

[12] Walberg J. L., Mole P. A., Stern J. S. (1982). Effect of swim training on development of obesity in the genetically obese rat. *American Journal of Physiology*.

[13] Ali A. S., Ali S., Ahmad A., Bao B., Philip P. A., Sarkar F. H. (2011). Expression of microRNAs: potential molecular link between obesity, diabetes and cancer. *Obesity Reviews*.

[14] Andersen N. N., Jess T. (2014). Risk of cardiovascular disease in inflammatory bowel disease. *World Journal of Gastrointestinal Pathophysiology*.

[15] Viboolvorakul S., Patumraj S. (2014). Exercise training could improve age-related changes in cerebral blood flow and capillary vascularity through the upregulation of VEGF and eNOS. *BioMed Research International*.

[16] Eisenstein R. (1991). Angiogenesis in arteries: review. *Pharmacology & Therapeutics*.

[17] Fernandes T., Magalhães F. C., Roque F. R., Phillips M. I., Oliveira E. M. (2012). Exercise training prevents the microvascular rarefaction in hypertension balancing angiogenic and apoptotic factors: role of microRNAs-16, -21, and -126. *Hypertension*.

[18] Fish J. E., Santoro M. M., Morton S. U. (2008). miR-126 regulates angiogenic signaling and vascular integrity. *Developmental Cell*.

[19] Da Silva N. D., Fernandes T., Soci U. P. R., Monteiro A. W. A., Phillips M. I., De Oliveira E. M. (2012). Swimming training in rats increases cardiac MicroRNA-126 expression and angiogenesis. *Medicine and Science in Sports and Exercise*.

[20] Medeiros A., Oliveira E. M., Gianolla R., Casarini D. E., Negrão C. E., Brum P. C. (2004). Swimming training increases cardiac vagal activity and induces cardiac hypertrophy in rats. *Brazilian Journal of Medical and Biological Research*.

[21] Scomparin D. X., Grassiolli S., Gomes R. M. (2011). Low-intensity swimming training after weaning improves glucose and lipid homeostasis in MSG hypothalamic obese mice. *Endocrine Research*.

[22] Chistiakov D. A., Orekhov A. N., Bobryshev Y. V. (2016). The role of miR-126 in embryonic angiogenesis, adult vascular homeostasis, and vascular repair and its alterations in atherosclerotic disease. *Journal of Molecular and Cellular Cardiology*.

[23] Rawal S., Munasinghe P. E., Shindikar A. (2017). Down-regulation of proangiogenic microRNA-126 and microRNA-132 are early modulators of diabetic cardiac microangiopathy. *Cardiovascular Research*.

[24] Neth P., Nazari-Jahantigh M., Schober A., Weber C. (2013). MicroRNAs in flow-dependent vascular remodelling. *Cardiovascular Research*.

[25] Baggish A. L., Park J., Min P.-K. (2014). Rapid upregulation and clearance of distinct circulating microRNAs after prolonged aerobic exercise. *Journal of Applied Physiology*.

[26] Pandya N. M., Dhalla N. S., Santani D. D. (2006). Angiogenesis-a new target for future therapy. *Vascular Pharmacology*.

[27] Prior B. M., Yang H. T., Terjung R. L. (2004). What makes vessels grow with exercise training?. *Journal of Applied Physiology*.

[28] Behn A., Ur E. (2006). The obesity epidemic and its cardiovascular consequences. *Current Opinion in Cardiology*.

[29] Brown M. D., Hudlicka O. (2003). Modulation of physiological angiogenesis in skeletal muscle by mechanical forces: involvement of VEGF and metalloproteinases. *Angiogenesis*.

